# Complications of Transfusion-Dependent β-Thalassemia Patients in Sistan and Baluchistan, South-East of Iran

**Published:** 2017-10-01

**Authors:** Maryam Yaghobi, Ebrahim Miri-Moghaddam, Naderi Majid, Ali Bazi, Ali Navidian, Asiyeh Kalkali

**Affiliations:** 1 Pregnancy Health Research Center, Zahedan University of Medical Sciences, Zahedan, Iran; 2 Genetics of Non-Communicable Diseases Research Centre, Zahedan University of Medical Sciences, Zahedan, Iran; 3 Cardiovascular Diseases Research Center, Birjand University of Medical Sciences, Birjand, Iran Clinical Research Development Unit, Amir-Al-Momenin Hospital, Zabol University of Medical Sciences, Zabol, Iran; 4 Department of Mental Health & Psychiatric Nursing, Pregnancy Health Research Centre, Zahedan University of Medical Sciences , Zahedan, Iran; 5 Students Scientific Research Center, Faculty of Nursing & Midwifery, Zahedan University of Medical Sciences , Iran Hospital, Iranshhar, Iran; 6 Students Scientific Research Center, Faculty of Nursing & Midwifery, Zahedan University of Medical Sciences , Iran Hospital, Iranshhar, Iran

**Keywords:** β- thalassemia major, Iron overload, Organ dysfunction, Cardiac disease

## Abstract

**Background**: Thalassemia syndromes are among prevalent hereditary disorders imposing high expenses on health-care system worldwide and in Iran. Organ failure represents a life-threatening challenge in transfusion- dependent β-thalassemia (TDT) patients. The purpose of the present study was to determine the frequency of organ dysfunctions among TDT patients in Sistan and Baluchistan province in South-East of Iran.

**Materials and Methods: **Laboratory and clinical data were extracted from medical records as well as by interviews. Standard criteria were applied to recognize cardiac, gonadal, endocrine and renal dysfunctions. The collected data were analyzed using the SPSS statistics software (Ver.19).

**Results: **A total of 613 TDT patients (54.3% males and 45.7% females) were included in this study. The mean age of patients was 13.3 ±7.7 years old. Cardiac events comprised the most encountered complications (76.4%), following by hypogonadism (46.8%), parathyroid dysfunction (22%), thyroid abnormalities (8.3%), diabetes (7.8%) and renal disease (1.8%). Hypogonadism comprised the most identified complication in patient <15 years old, while the cardiac complications were the most frequent sequela in patients >15 years old (P<0.01).

**Conclusion: **As cardiac events are significantly more common among TDT patients, close monitoring of the heart function is recommended for identifying patients with cardiac problems.

## Introduction

 Transfusion-dependent β-thalassemia (TDT) is the most frequent form of severe congenital anemia, and comprises one of the most important health problems in Iran. Mutations responsible for various forms of TDT usually lead to non-productive β-globin gene ^1^^,^^2^ . Regular blood transfusion and continual administration of iron-chelators are conventional therapeutic strategies in TDT^   3 ^. 

Despite progressive advancements in developing various iron scavenging drugs, iron overload is still considered a challenging conundrum in these patients. Historically, organ dysfunctions resulting from deleterious effects of free iron have been leading causes of morbidity and mortality among TDT patients^   4 ^. Potentially, excess iron can adversely affect normal function of any organ; however, endocrinesystem, liver, heart and kidneys are primary targets. 

Sistan and Baluchistan province, which is located in a sub-tropical area in the South-East of Iran, harbors as high as 10 % of β-thalassemia carrier population (in comparison with 4-8% of the carriers in other regions of Iran) 5^,^^6^ . There are also 2500 identified TDT patients in Sistan and Balouchestan province^   7 ^. This large population raises concerns regarding psychological and financial burden of forthcoming complications. Despite this, no studies have been carried out on the prevalence of transfusion-related complications in TDT patients in the province. In this descriptive cross-sectional study, we evaluated the frequencies of cardiac, endocrine as well as renal dysfunctions in TDT patients admitted to Ali-Asghar Hospital, Zahedan, Sistan and Baluchistan.

## MATERIALS AND METHODS

 The present study was carried out on 613 TDT patients (diagnosed based on cell blood counts, hemoglobin (Hb) electrophoresis and clinical findings) admitted to Ali-Asghar Hospital, Zahedan, Sistan and Baluchistan, in 2014. The study was approved by Ethics Committee of Zahedan University of Medical Sciences. Patients or their parents signed informed consent forms during interviews.

Demographic data (sex, age, weight, height, transfusion intervals as well as Hb and ferritin levels) were collected from interviews and medical archives.Transfusion-related complications (endocrine system, cardiac and renal functions) were assessed using available laboratory and clinical histories of the patients. The diagnosis of organ dysfunctions was based on supporting laboratory results as well as confirmatory clinical evidences.

Failure to sexual thrive was defined based on the Marsha-Tanner et al. criteria, and was regarded as a hypogonadal sign^   8 ^. Other hypogonadism criteria were insufficient growth of secondary sexual organs in boys and girls at the maturity age (13 and 14 years old for girls and boys, respectively) and low sexual hormone levels (luteinizing hormone (LH) and Follicle-stimulating hormone (FSH) for females and testosterone for males). Thyroid function was assessed by evaluating the levels of thyroid hormones (Thyroxine (T4) and Thyroid-stimulating hormone (TSH)). Hypothyroidism was established in patients with TSH >5 mlU/L and T4 <5.4 μg/dl. Parathyroid function was judged by the serum Calcium (Ca), phosphate (P) and parathyroid hormone (PTH) values. Hypoparathyroidism was considered in cases with low Ca, high P and low PTH levels. The diagnosis of diabetes mellitus (DM) was established in patients who were under insulin therapy. Also, individuals with FBS level above 126 mg/dl or occasional blood sugar above 200 mg/dl were considered as DM. Cardiomyopathy (Cmp) and reduced diastolic and systolic volumes were regarded in patients with abnormal electrocardiography results. Finally, renal dysfunction was defined based on abnormal serum blood urea nitrogen (BUN) and creatinine levels as well as glomerular filtration rate (GFR) according to age.

## Results

 Of the total TDT patients (n=613) studied, 333 (54.3%) were males and 280 (45.7%) were females. Mean age was 13.3 ±7.7 years old. The majority of the patients (385 cases, 62.8%) had age< 15 years old, 191 (31.2%) were 15-25 years old, and 37 (6%) were >26 years old. Table 1 represents demographic and laboratory characteristics of the patients. 

**Table 1:** Demographic and laboratory data of 613 transfusion-dependent 𝜷-thalassemia patients

**Characteristic**	**Sex**	**Mean value**	**SD**
Age (years)	M F	13.8 12.7	7.61 7.85
Mean weight (Kg)	M F	32.1 28.7	15.18 13.23
Mean height (cm)	M F	134 126	25.3 23.7
BMI (Kg/m^2^)	M F	17.3 17.1	3.56 3.87
Volume of transfused Blood (ml)	M F	481.6 452.8	159.86 165.24
Mean transfusion intervals	M F	22.8 24.1	3.7 4.1
Hemoglobin (g/dl)^a^	M F	9.4 9.5	0.72 0.66
Ferritin (ng/ml)^a^	M F	3685 3939	2319 2116

a ; Measurement at the three last occasions.

Cardiac complications were the most common morbidities with the rate of 76.4% (244/390). Other common complications included hypogonadism (46.8%, 148/316), parathyroid dysfunctions (22.1%, 63/285), thyroid abnormalities (8.3%, 30/356), DM (7.8%, 48/613) and renal problems (1.8%, 8/434). Hypogonadism was the most commonly encountered complication in patients <15 years old (86.5%), while cardiac abnormalities were observed most commonly in cases >15 years old (81.4% in patients 15-26 years old and 87.5% in patients>26 years old (P<0.01, Table 2).

**Table 2:** The frequency of complications observed in transfusion- dependent 𝜷-thalassemia patients among three age groups

**Complications**	**Evaluated patients** **(N)** *	**Observed abnormal ** **functions**	**Age Groups (years)**			
		**N (%)**				
			<15 N(%)	15-26 N(%)		>26 N(%)
Cardiac Complication's	390(a)	244(76.4)	94(44.9)	122(81.4)		28(87.5)
Hypogonadism	316(b)	148(46.8)	77(86.5)	60(31.5)		11(29.7)
Hyperparathyroidism	285(c)	38(13.3)	18(15.3)	20(14.9)		0(0)
Hypoparathyroidism	285(c)	25(8.7)	7(5.9)	12(8.9		6(16.2)
Hypothyroidism	356(d)	28(7.8)	11(6.8)	15(9.3)		2(5.5)
Hyperthyroidism	356(d)	2(0.5)	0(0)	2(1.2)		0(0)
Diabetes	613(e)	48(7.8)	8(2)	26(13.6)	14(37.8)	
Renal	434(f)	8(1.8)	5(1.8)	2(1.4)		1(3.5)

* : Respective number of patients in age groups of <15 y, 15-25 y and >26 y are as following.

(a) ; 207, 151, 32,

(b) ; 89, 190, 37,

(c) ; 385, 191, 37,

(d) ; 117, 134, 34,

(e) 160, 160, 36,

(f) ; 271, 135, 28.

Cardiomyopathy (Cmp) was the most cardiac condition (71.7%), while the leastof the patients with abnormal echocardiography findings represented with reduced systolic volume (S.v) (1.2%, Table 3). Overall, Cmp also comprised the most observed sequel in males (78.7%) and females (63.9%). Table 4 shows the frequencies of various cardiac complications in three age groups in TDT patients

**Table 3:** The distribution of transfusion-dependent 𝜷-thalassemia complications in males and females

**Complication** *			**Male N (%)**	**Female N (%)**
Cardiac Complications(a)		Cmp	104(78.8)	71(63.9)
		Reduced D.v	21(15.9)	39(35.2)
		Reduced S.v	3(2.3)	0(0)
		Reduced D.v	4(3)	1(0.9)
Hypogonadism (b)	No sexual maturity		60 (12.7)	88(61.2)
	Normal Sexual Maturity		113(24)	55(38.2)
	Not mature age		296(63.3)	1(0.6)
Parathyroid (c)	Hypoparathyroidism		15(9.8)	10(7.5)
	Normal		124(81)	98(74.3)
	Hyperparathyroidism		14(9.2)	24(18.2)
Thyroid (d)	Hypothyroidism		13 (6.7)	15(9.3)
	Normal		178 (92.3)	148(90.7)
	Hyperthyroidism		2 (1)	0(0)
Diabetes (e)	Diabetes		26 (7.8)	22(7.8)
	No diabetes		307(92.2)	258(92.2)
Renal function(f)	Abnormal		7(2.9)	1(0.5)
	Normal		229(97.1)	197(99.5)

Abbreviations: cmp; cardiomyopathy; S.v; Systolic volume, D.v; Diastolic volume.

* ; Respective total numbers of male and female patients are:

(a) ; 132, 111,

(b) ; 469, 144,

(c) ; 153, 132,

(d) ; 193, 163,

(e) ; 333, 280,

(f) ; 236, 198.

**Table 4:** The frequency of cardiac complications identified in three age groups oftransfusion-dependent 𝜷-thalassemia patients

**Cardiac event**	**<15 years (N=94)** **n (%)**	**16-25 years (N=122)** **n (%)**	**>26 years (N=27)** **n (%)**	**Total (N=243)** **n (%)**
Cmp	75 (79.8)	86(70.5)	14(51.8)	175(72.1)
Reduced D. v	16(17)	33(27)	11(40.8)	60(24.7)
Reduced S. v	1(1)	1(0.8)	1(3.7)	3(1.2)
Reduce D. v & S. v	2(2.2)	2(1.7)	1(3.7)	5(2)

Cmp; Cardiomyopathy, D. v; Diastolic volume, S. v ; Systolic volume.

## Discussion

 Excess iron can potentially participate in any vital organ which subsequently may lead to organ dysfunction^   4 ^. Regarding the high susceptibility of cardiomyocytes to iron toxicity, heart failure comprises a potentially common life-threatening event in TDT patients^   9 ^. Overall, the abnormal electrocardiogram (ECG) findingswere the most common encountered abnormality in our patients (76.4%). In addition, cardiac diseases encompassed as the main problem in patients >15 years old. In previous studies in Iran, cardiac abnormalities comprised 9-33% of thalassemia complications  ^10^^, ^^11^ , which is significantly lower than our results. This may be related to higher ferritin levels in our patients. In fact, themajority of our patients had poor compliance with iron chelating drugs. In addition, cardiac disorders were encountered in lower age groups in our study in comparison to otheronesconducted on Iranian patients ^12^^,^^13^ . It is noteworthy to emphasize that heart failure is the major cause of death in older TDT patients. In order to prevent imminent threats, it is highly recommended to routinely screen the heart function in TDT patients. 

Hypogonadism was the second most common complication and themost common endocrinopathy identified in our patients. A largepercentage (86.5%) of the patients <15 years old had signs of hypogonadism, raising concerns about reproductive abilities of these patients in young adulthood. In a study by Albu et al., 54.1% of TDT patients showed signs of hypogonadism^   14 ^. In addition, we noticed that among patient who reached puberty age (173 boys and 143 girls), 34 % of males and 61% of females failed to attain normal sexual development which highlights hypogonadism as a more serious issuein females. Early occurrence of hypogonadism in our patients may be due to poor compliance with iron chelation therapy in our patients which in turn cause iron deposition in endocrine organs and puberty failure. 

Parathyroid gland insufficiency was the third most common adverse complication (63/285, 22%) observed in our patients. Hyperparathyroidism was detected in a higher ratio (38/285, 13.3%) than hypoparathyroidism (25/285, 8.7%). Parathyroid dysfunction has been described in varying ratios of 1-40% in different reports ^15^^-^^17^ . In a study by Isik et al, hyperparathyroidism was reported in 11.5% of TDT patients^   18 ^.Moreover, hypoparathyroidism has been observed in 8.5-11.1% in other studies ^19^^, ^^20^ . These results are similar to the ratios observed in thecurrent study. Nevertheless, routine examination of parathyroid function is necessary to early diagnosis this condition. 

We identified thyroid dysfunction in 8.3% (30/356) of our patients; of whom 28hadhypothyroidism.In a study conducted by Isik et al., the frequency of hypothyroidism among TDT patients was 6.1% ^   18 ^. In other studies, hypothyroidism  was reported in 3.5% of TDT patients ^10^^,^^20^ .Thyroid abnormal activity constitutes a prevalent complication in TDT necessitating early diagnosis.

In the current study, the frequency of DM reached 7.8 % (48/613). In contrast to previous discussed complications, DM was a feature found more frequently among young adults than adolescents (51.4 % in patients >15 years old and only 2% in patients <15 years old). In others centers, DM occurrence has been reported in 6-30% of TDTpatients^15^^-^^17^^,^^21^^,^^22^. Nevertheless, DM is a serious complication requiring regular screening for early identification.

In the present study, renal dysfunction was identified in 1.8% of TDT patients (2.9% males and 0.5% females). Some studies have described abnormal renal function as a common complication in TDT patients ^23^^,^^24^; however, no cases of renal dysfunction were detected in astudyon 340 TDT patients conducted by Vahidi et al. ^   20 ^. Besides, some authors have questionedthe sensitivity of current tests for diagnosis of renal disease in TDT^   10 ^. Developing more sensitive markers for detection of renal involvement may reveal more cases with renal abnormalities in TDT patients.

## CONCLUSION

 A high ratio of cardiac abnormalities was observed in our TDT patients reflecting less effective iron management strategies.We recommend providing high-sensitive imaging facilities for TDT patients to detect at-risk groups for heart dysfunction. The results also indicated alower age of onset for different complications among our participants. Therefore, regular screening of the patients in lower ages is highly recommended for early identification and management of different morbidities.
